# Effects of mine water on growth characteristics of ryegrass and soil matrix properties

**DOI:** 10.1038/s41598-022-22625-y

**Published:** 2022-10-22

**Authors:** Lianman Xu, Linlin Du, Yajing Li, Weizhe Li, Hasa Wu

**Affiliations:** Liaoning University, Shenyang, China

**Keywords:** Ecology, Environmental sciences

## Abstract

Irrigation with mine water not only improves water resource utilization rates and alleviates water shortages but can also promote crop growth and yields. However, long-term irrigation with mine water can significantly change the physicochemical properties of soil due to its unique mineral content. In this study, two groups of experiments were conducted (pot experiments and soilless cultivation) using mine water from the Fushun mining area to explore its effects on the physiological and photosynthetic characteristics of ryegrass, as well as soil properties. Mine water irrigation inhibited all of the indicators evaluated in this study, whereas a mixture of clean water and mine water had a stimulatory effect. Interestingly, this stimulatory effect was weakened as the proportion of mine water increased but reached its maximum when the ratio was 2:1. Moreover, the inhibitory effect of the irrigation water was weakened as the proportion of clean water increased. The contents of K^+^, Na^+^, Ca^2+^ and Mg^2+^ in soil were higher than those in the soil matrix, and the content of the nutrient elements N, P and K, and metal cations increased gradually as the mine water ratio increased, and the electrical conductivity increased significantly. Moreover, the pH of the soil decreased steadily (i.e. acidity increased) with increased soil salinity. Our findings indicated that a mixture of mine water from Fushun mining area and clean water at a 1:2 ratio could improve the physiological, growth, and photosynthetic characteristics of ryegrass by enhancing soil quality. Our study thus provides an experimental precedent for the utilization of mine water in ecological restoration and agricultural irrigation, and could therefore serve as a basis for the development of novel strategies for environmental restoration and the utilization of water resources.

## Introduction

Although industrial effluents are a nutrient-rich and high volume source of water, they are generally considered unsuitable for crop irrigation because of the presence of various chemicals, limiting the potential for reuse. If high quality industrial wastewater or treated water is used for agricultural irrigation, the water shortage will be significantly alleviated^1^. Water shortages have become a serious problem in China in recent years^2^. However, up to 4.2 billion m^3^ of water are discharged from coal mines every year, with utilization rates often being as low as 25%^3^. Coal has always been an important energy source, and the amount of mining and demand are increasing. Water is indispensable in the mining process. The amount of groundwater exposed during mining and the amount of water used in coal production is enormous. If this kind of water (mine water) is reused, the implications will be significant. This not only constitutes a waste of large volumes of available water resources, but also poses a threat to the mining area and the surrounding environment^4–7^. Although mine water is generally considered a pollution source, it is also a precious water resource^8–11^. When mine water is mixed with clean water for irrigation, it not only promotes plant growth of but also reduces water waste and prevents the pollution of soil and water caused by mine water discharge. However, mine water not only contains mineral elements that benefit plant growth but also high salt contents and heavy metals, and can therefore have a negative impact on plant growth and quality when used for long-term irrigation^12–14^. Therefore, additional studies are needed to explore the optimal clean water/mine water irrigation ratio, as well as to develop novel mine water irrigation strategies.

Several studies have characterized the effects of reclaimed water on crop growth and soil properties when used in agricultural irrigation. Chuncheng Liu et al. reported that irrigating with a 1:1 mixture of brackish water and reclaimed water did not significantly affect the moisture and salt contents of soil, nor did it affect the physiological index and biomass of *Brassica chinensis* in pot experiments. These findings suggested that clean water could be safely replaced with a combination of reclaimed water and brackish water for irrigation^15^. Xue Gong et al. found that reclaimed water irrigation could increase soil organic matter and total nitrogen content, and concluded that it could increase soil fertility to a certain extent^16^. Similarly, Jian Wang reported that soil structure and the yield and quality of winter wheat were improved when mixed irrigation was administered at an appropriate ratio^17^. Shipei Gong found that mine water improved soil characteristics in a desert ecosystem and promoted vegetation growth^18^. Anita Singh et al. evaluated the risk of several heavy metals to human health in vegetables, cereal crops, and milk produced using mine water irrigation. The authors reported that wastewater irrigation led to an accumulation of heavy metals in food, and heavy metal pollution in sewage irrigation sites pose a serious threat to human health^19^.

Several studies have characterized the effects of crop irrigation using sewage and reclaimed water on crop growth, soil characteristics, and groundwater quality. However, very few studies have explored the applicability of mine water mixed with clean water in agricultural irrigation^20^. Therefore, our study cultivated ryegrass, both in pot experiments and through soilless cultivation, and irrigated the ryegrass with different ratios of mine water and clean water. Furthermore, our study explored the effects of the ratio of mine water and clean water on the germination potential and germination rate of ryegrass seeds, soluble sugar, soluble protein, net photosynthetic rate, transpiration rate, intercellular CO_2_ concentration, and stomatal conductance. Our study also explored the effects of mine water irrigation on soil physical and chemical properties. Finally, we also comprehensively analyzed the influence of mine water irrigation on plant growth and soil properties to determine the optimal clean water to mine water ratio. Collectively, our findings provide useful insights for the development of efficient utilization mine water strategies, as well as for agricultural irrigation and ecological restoration.

## Materials and methods

### Experiment materials

The mine water was obtained from the Laohutai Mine in Shenyang, Liaoning Province, after which it was sealed and transported to the laboratory for later use. Table 1 summarizes the mine water chemical test results. All plant growth experiments were conducted using ryegrass (ryegrass seeds were purchased from DLF-TRIFOLIUM). Shallow soil with a depth of 20 cm in the mine area was used as the matrix for ryegrass planting, and the properties of soil are shown in Table 2.

**Table 1:** Main components of mine water in the Fushun mining area.

pH	The content of metal element (± s.d.) (mg/L)							
	K	Ca	Mg	Na	Al	Fe	Cr	Pb
6.76 ± 0.077	73.2 ± 0.083	110.5 ± 0.515	75.3 ± 0.637	42.5 ± 0.668	0.06 ± 0.007	0.72 ± 0.033	0.26 ± 0.057	0.08 ± 0.008

**Table 2:** Properties and composition of the soil in the Fushun Mining area.

pH	The content of ion and elemental (± s.d.) (mg/L)						
	K^+^	Ca^2+^	Mg^2+^	Na^+^	N	P	K
7.23 ± 0.077	25.3 ± 0.121	53.2 ± 0.416	76.3 ± 0.587	21.6 ± 0.613	32.16 ± 0.545	9.84 ± 0.156	78.21 ± 0.356

### Experiment process

The experiments were conducted in Petri dishes with a diameter of 15 cm and a height of 3 cm. Approximately 2 cm of fine quartz sand was spread evenly in the dish to germinate the seeds and conduct soilless cultivation. In the pot experiments that used soil, the indices of soil base samples were measured first. Mine water was mixed with clean water according to the ratios in Table 3.

**Table 3:** Proportions of mine water and clean water in different groups.

Seed germination			Soilless cultivation			Pot experiments		
Group	Clean water	Mine water	Group	Clean water	Mine water	Group	Clean water	Mine water
T1	1	0	A1	1	0	B1	1	0
T2	2	1	A2	2	1	B2	2	1
T3	1	1	A3	1	1	B3	1	1
T4	1	2	A4	0	1	B4	0	1
T5	0	1						

Similarly-sized ryegrass seeds were selected, soaked, and disinfected with sodium hydroxide solution, washed several times, and dried with a filter paper. A total of 100 seeds were germinated in each group in an artificial climate incubator. Germination potential was calculated on the 7th day and germination rate was calculated on the 14th day. Germination was monitored and recorded for 15 consecutive days. Upon reaching a 5–6 leave stage, the seedlings were irrigated 3–4 times a week with different proportions of mine water ensuring that the amount of water was appropriate. On the 20th day, the penultimate full-spread functional leaf of the ryegrass plants was selected to measure its physiological indices and photosynthetic characteristics. In the pot experiments, the physiological characteristics, growth characteristics and photosynthetic effect of the plant were measured. Characterizations were conducted using 1 cm depth soil from the middle layer of each group, which was crushed and sieved with a 1 mm screen.

### Germination method

Seed germination is the first phase of plant growth and development. Germination rate and germination potential are important indices to measure the germination ability of seeds, which can reflect seed germination number and germination speed. During seed germination, the number of germinated seeds was recorded every day, and the germination potential and germination rate were calculated according to the following formula.

$${\text{Germinated}}\,{\text{potential}}\, = \,{\text{Number}}\,{\text{of}}\,{\text{germinated}}\,{\text{seeds}}\,{\text{within}}\,{7}\,{\text{days}}/{\text{Total}}\,{\text{number}}\,{\text{of}}\,{\text{seeds}}\,{\text{tested}}\, \times \,100\% .$$

$${\text{Germinated}}\,{\text{rate}}\, = \,{\text{Number}}\,{\text{of}}\,{\text{normal}}\,{\text{seedlings}}\,{\text{within}}\,{14}\,{\text{days}}/{\text{Total}}\,{\text{number}}\,{\text{of}}\,{\text{seeds}}\,{\text{tested}}\, \times \,100\% .$$

The content of soluble sugar and soluble protein can reflect the resistance of the plant (i.e. higher sugar contents are linked to more resistant plants). Therefore, both of the contents of soluble sugar and soluble protein in ryegrass leaves were determined.

The sugar content was determined by anthrone colorimetry^21^. A certain number of ryegrass leaves were placed in an oven and dried at 110 °C for 15 min. Afterward, the leaves were further dried at 70 °C to a constant weight. Next, 0.05 g of dried leaves were ground and placed in test tubes, to which 15 ml of distilled water was added. The samples were then boiled for 20 min, allowed to cool, and filtered through a funnel with activated carbon. The filtrate was then transferred to a 100 ml volumetric bottle. Next, 1 ml of extract was transferred to a dry test tube, and 5 ml of anthrone reagent was added. The sample was then boiled for 10 min, allowed to cool, and its absorbance was measured at 620 nm using a spectrophotometer. Finally, the soluble sugar contents of the ryegrass plants were calculated.

The protein content was determined by Coomassie bright blue G-250 staining. First, 0.1 g of fresh plant sample was added to 2 ml of distilled water, followed by a small amount of quartz sand for homogenization. The homogenate was then mixed with a washing solution in centrifuge tubes. Next, the samples were centrifuged for 10 min at 4000 r·min^−1^, after which the supernatant was transferred to a 10 mL volumetric bottle. Afterward, 0.1 ml of extract was transferred to a clean test tube and 5 ml of Coomassie bright blue reagent was added. The mixture was then shaken and allowed to sit undisturbed, after which the absorbance at 595 nm was measured with a spectrophotometer. The protein content of the samples was then determined using a standard curve. Finally, the soluble protein content of the ryegrass plants was calculated.

Photosynthetic characteristics can reflect plant growth and quality. Therefore, we measured the photosynthetic characteristics of plants using a portable photosynthetic apparatus. The artificial light source was selected as follows: temperature, 25 ± 0.5 °C; external CO_2_ concentration, 380 μmol·mol^−1^; flow rate, 400 μmol·s^−1^; relative humidity, 60%; illumination intensity, 1000 μmol·m^−2^·s^−1^. The penultimate fully functional leaf of the ryegrass plant was selected to measure the net photosynthetic rate, transpiration rate, intercellular CO_2_, and stomatal conductance from 9 to 11 am on sunny days. The average value of five plants was determined each time, and each treatment was repeated three times.

Next, 5 g of soil sample were transferred to a shock tube, to which 20 ml of distilled water was added. The mixture was then shaken and centrifuged for 15 min. The supernatant was then recovered and allowed to sit undisturbed. The content of alkali-hydrolyzed nitrogen in soil was determined via the alkali-hydrolyzed diffusion method. NaHCO_3_ was used to determine the content of soil available phosphorus. The content of available potassium in soil was determined via NH_4_OAc extraction-flame photometry. All experiments and field studies described herein were compliant with relevant institutional, national, and international guidelines and legislation.

The soil sample was then placed in the incubator to air dry to constant weight. Next, 50 g of dry soil sample was placed in a conical flask (500 mL) add distilled water was added to achieve a soil-to-water mass ratio of 5:1. The samples were then thoroughly mixed and shaken. The liquid was filtered with filter paper, and a clear filtrate was collected. Its physical and chemical properties were then determined. A PHBJ-260 portable pH meter was used to determine the pH of the soil extract. The conductivity of the soil extract was measured with a DDS-307 conductivity meter. The contents of water-soluble Ca^2+^ and Mg^2+^ ions in the soil were determined via EDTA titration. The content of K^+^ and Na^+^ in the soil extract was determined by FP6400 flame photometry. In the study, the number of test samples for soil, mine water and ryegrass indexes was three, and the average value was taken after measurement.

## Results and analysis

### Effects of mine water on seed germination

**Figure 1:**
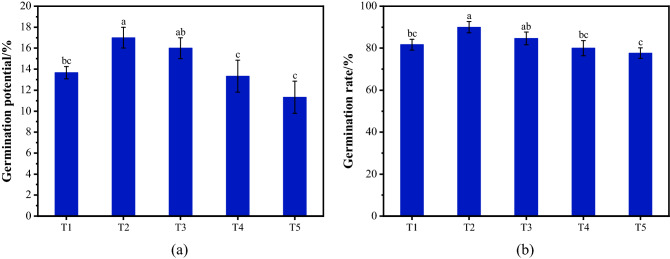
Effects of mine water on seed germination (One-Way ANOVA, *α* = 0.05). (**a**) Germination potential; (**b**) the germination rate.

The influence of mine water on seed germination is shown in Fig. 1. As illustrated in the figure, the influence of mine water proportion on seed germination potential and germination exhibited similar trends. With increasing mine water proportion, the germination rate and germination potential showed a decreasing trend, and reached their maximum when the ratio was 2:1 (T2). In T2, the germination potential and rate were 17.1 and 89%, and in T5 were 11.3 and 78%, respectively. The results of Significance test are shown in the figure, there were significant differences between T2 and T1, T4 and T5 (*P* < 0.05), but no significant differences between T2 andT3. The difference of germination rate between groups was greater than germination potential. It indicated that the effect of soaking seeds in mine water on germination potential was more obvious than germination rate. Therefore, we concluded that soaking the seeds in mine water from the Fushun mine area prolonged the germination time and did not significantly affect the germination rate.

### Effects of mine water on physiological characteristics

**Figure 2:**
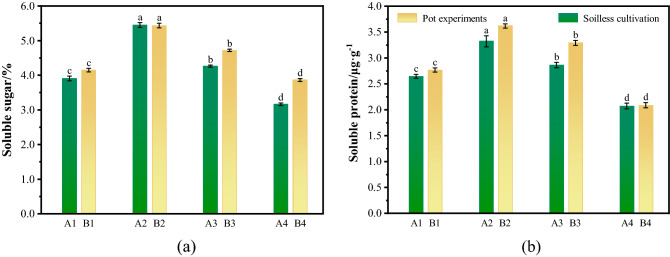
Effects of mine water on plant physiological characteristics (One-Way ANOVA, *α* = 0.05). (**a**) Soluble sugar; (**b**) soluble protein.

The ryegrass was watered with mine water mixed with clear water in different proportions, which had different effects on the resistance indexes of ryegrass.Fig. 2 illustrates the influence of mine water irrigation on the physiological characteristics of ryegrass. As shown in Fig. 2a, the soluble sugar content decreased with higher mine water proportions, and the soluble sugar content reached a minimum when the seedlings were exclusively irrigated with mine water. When the ratio of mine water to clean water was 1:2, the content of soluble sugar reached its maximum (5.43%). Compared with the control group, the soluble sugar content increased by 30.8% in the pot experiments (with soil) and 39.4% in the soilless cultivation experiments. As the proportion of mine water increased, the content of soluble sugar decreased gradually. Afterward, with the increase of water proportion, the soluble sugar content did not increase, but showed a decreasing trend. When irrigated only with mine water, the content of soluble sugar was the lowest, and the content under soilless cultivation was lower than that under soilless cultivation (3.16%), which was reduced by 19.2% compared with A1 and 42% compared with A2 (*P* < 0.01). As shown in Fig. 2b, the soluble protein content decreased with increasing mine water proportion, and the content of soluble protein reached its maximum when the ratio of clean water to mine water was 2:1. Protein content was lowest when the plants were exclusively irrigated with mine water, which decreased by approximately 23% compared with the control group and approximately 42.4% compared with the maximum of B2 (*P* < 0.01).

### Effects of mine water on photosynthetic characteristics

**Figure 3:**
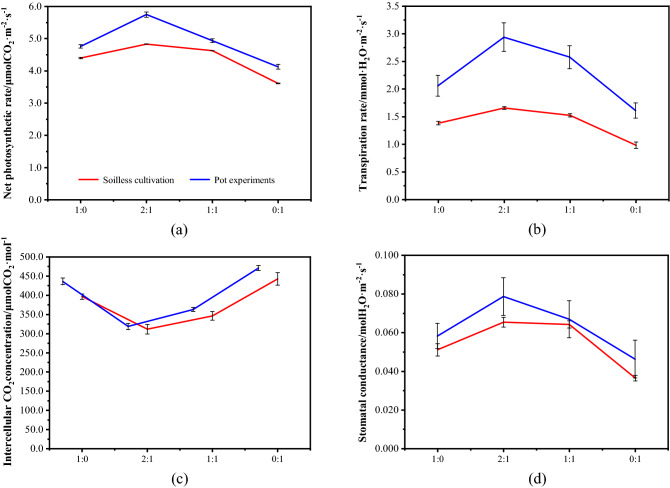
Effects of mine water on photosynthetic characteristics. (**a**) Net photosynthetic rate; (**b**) transpiration rate; (**c**) intercellular CO_2_ concentration; (**d**) stomatal conductance.

The influence of mine water on the photosynthetic characteristics of ryegrass is shown in Fig. 3. The net photosynthetic rate, transpiration rate, and stomatal conductance exhibited the same variation trends with the change of mine water proportion, while the intercellular CO_2_ concentration showed the opposite trend. When the mine water ratio declined, all of net photosynthetic rate, transpiration rate, and stomatal conductance decreased. When the ratio of clean water to mine water is 2:1 (A2 and B2), the net photosynthetic rate, transpiration rate, and stomatal conductance reach the maximum value. When irrigating with only mine water (A1 and B1), all indicators were the lowest, reaching values that were lower than the indicators of the clean water control group. The intercellular CO_2_ concentration was lowest in A2 and B2, then it increased with the proportion of mine water. Moreover, the photosynthetic effect of soil pot was stronger than that of soilless cultivation.

As shown in Fig. 3a, the net photosynthetic rate of leaves in group B2 was the highest, which was 5.74 μmol CO_2_ m^−2^ s^−1^. As the proportion of mine water increased, the net photosynthetic rate decreased gradually. The net photosynthetic rate of the B4 group was the lowest (4.12 μmol CO_2_ m^−2^ s^−1^), which was 13.3% lower than that of B1 and 28.2% lower than that of B2. As shown in Fig. 3b, the transpiration rate decreased with the increase of mine water proportion. The highest transpiration rate of group B2 was 2.94 mmol H_2_O m^−2^ s^−1^, which was 42.7% higher than that of B1 and 82.6% higher than that of B4. B4 had the lowest transpiration rate, which was 21.8% lower than that of B1. As shown in Fig. 3d, the variation of stomatal conductance with the proportion of mine water was similar to that of the net photosynthetic rate and transpiration rate. B2 reached a maximum of 0.079 mol H_2_O m^−2^ s^−1^, which was 36.2% higher than the value observed in the B1 group. B4 reached a minimum of 0.046 mol H_2_O m^−2^ s^−1^, which represented a 20.7% decrease compared with B1 and 41.8% compared with B2. As illustrated in Fig. 3c, the intercellular CO_2_ concentration reached the lowest point in B2, and the maximum point was 466 μmol CO_2_·mol^−1^ in B4, which was 20.7% lower than B1 and 41.8% lower than B2.

### Effects of mine water on soil matrix characteristics

**Figure 4:**
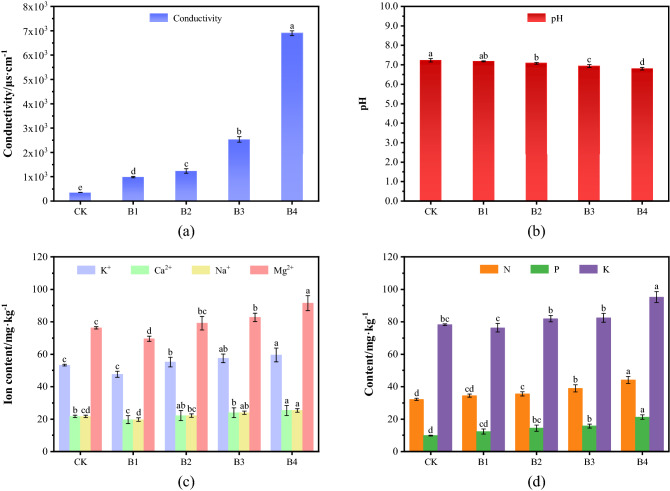
Effects of mine water on physical properties of soil (One-Way ANOVA, *α* = 0.05). (**a**) Changes in conductivity of soil; (**b**) change in pH of soil; (**c**) changes in the ion content of soil; (**d**) change in nutrient elements of soil.

The effects of mine water on the physical properties of the soil matrix are shown in Fig. 4. As illustrated in Fig. 4a, the conductivity of the B1–B4 soil samples was significantly higher than that of the soil base sample, and the conductivity of the soil samples increased markedly as the proportion of mine water increased (*P* < 0.01). The conductivity of B1 was 985.6 μs/cm, which was approximately 2.8 times that of the soil matrix. The conductivity of B4 reached the maximum point of 6897.5 μs/cm, which was approximately 20 times that of the soil matrix. In contrast, the changes in the pH of the soil samples were relatively small. Concretely, soil acidity increased gradually (i.e. pH decreased gradually from 7.23 to 6.81) when the mine water proportion increased (*P* < 0.05). As illustrated in Fig. 4c, the concentration of K^+^, Na^+^, Ca^2+^ and Mg^2+^ in soil was lowest when mine water was exclusively used for irrigation. Generally, with the increase of mine water proportion, the content of four kinds of ions increases continuously, B4 exhibiting the highest metal ion contents. The metal ion contents of the B4 group were 31.4, 25.3, 59.6 and 91.4 mg/kg, which represented a 24.1%, 17.1%, 12% and 19.8% increase compared with the basal sample, respectively, and increased by 33.1%, 28.4%, 25.2% and 31.5% compared with B1. Under the condition B1, the content of four major metal ions in the soil after irrigation had the smallest change compared with the content in the soil matrix before the experiment.

The effects of mine water irrigation on the nutrient elements of the soil are shown in Fig. 4d. Particularly, the contents of alkali-hydrolyzed nitrogen, available phosphorus, and available potassium in the soil matrix were the lowest. After planting the ryegrass, the contents of nutrient elements were increased in different degrees, and the contents of three nutrient elements in the water control group were slightly higher than in the soil matrix, reaching values of 34.46, 12.37 and 76.32 mg/kg, respectively. With increasing mine water ratios, the contents of all three elements in the soil matrix tended to increase. The maximum value was observed in the B4 group, with levels that were 37.4%, 116% and 21.8% higher than in the soil matrix. When the ratio of clean water to mine water was 2:1 (B2), the content of available phosphorus increased by approximately 46.6%, alkaline hydrolyzable nitrogen increased approximately 10.6%, and available potassium increased by 4.68%.The total amount of N, P and K in the soil before planting the ryegrass was 120.2 mg/kg. However, the total amount of nutrient elements increased when the ratio of clear water increased. In contrast, the total amount of nutrient elements was 123.15 mg/kg when the samples were irrigated with clean water, which had little change compared with pre-planting. After irrigation with only mine water, the total amount of nutrient elements reached up to 160.64 mg/kg, which represented an 33.64% increase compared with the matrix and 30.4% compared with the B1 group.

## Discussion

Our findings indicated that mine water had a certain inhibitory effect on ryegrass seed germination, and the intensity of this inhibitory effect increased with increased mine water proportion. These effects were mainly reflected as changes in germination potential. Concretely, irrigation with mine water prolonged the germination of ryegrass seeds but had no significant effect on germination rate. Min Zhu et al. found that recycled water inhibited the seed germination of turfgrass, and this effect became more notorious when the concentration of reclaimed water increased. This was likely because the water contained salt ions, heavy metal ions, and *E. coli*, all of which are known to affect seed germination^22^. The mine water was taken from the Laohutai mining area, where the water composition and quality are good. Therefore, mine water did not significantly affect seed germination and the seeds maybe germinate normally if given sufficient time.

The physiological and photosynthetic characteristics of ryegrass were impacted by the mine water, and the intensity of inhibition increased with higher mine water proportions. When the ratio of mine water to clean water reached a certain proportion (1:2, A1 and B1), the physiological and growth characteristics of ryegrass were improved to a certain extent. When only mine water was used for irrigation, the indices were significantly suppressed. In contrast, mixing clear water with mine water for irrigation promoted the physiological characteristics of the plants, as well as photosynthesis. This was likely because the mineral content of mine water is higher. However, mine water not only contains elements needed for plant growth but also some elements and ions that have inhibitory effects on plant growth. Therefore, the quality of ryegrass growth were suppressed when irrigating only with mine water. In contrast, after mixing the mine water with clean water, the concentrations of certain substances that produce adverse effects are diluted, and the mixed irrigation water promoted ryegrass growth in appropriate proportion.

A certain concentration of heavy metal elements will affect the absorption of essential elements by plants and produce antagonism, and high concentration can directly lead to plant death. Heavy metal stress affects chlorophyll content through two aspects: Heavy metal destroys enzymes needed for chlorophyll synthesis, affects plant chlorophyll synthesis, and then inhibits plant photosynthesis^23^. The second is the destruction of chloroplast structure and cell membrane^24–26^. In the treatment of high concentrations of heavy metals, the chlorophyll content of plants is significantly reduced due to the inhibition of chlorophylase or aminolevulinic acid dehydrase, thus inhibiting plant photosynthesis^27^. The heavy metal threat forcing stimulates the formation of reactive oxygen species that convert fatty acids into toxic lipid peroxides, which damage to plant cells^28–30^. Heavy metal stress can induce a lot of activity in plants sexual oxygen and inhibit the normal metabolism of plants, causing membrane lipid peroxidation and increased plasma membrane permeability^31, 32^. Low concentration of heavy metal stress will stimulate the protective mechanism of plants, so low concentration of stimulation will not damage plants, on the contrary, may help plant growth. Heavy metal stress causes water loss in plants, and a certain amount of proline can be produced to regulate the water balance of plant cells and reduce the damage degree of plant cells^33^. SOD, POD and CATT are important antioxidant enzymes in plants, which can scavenge excessive free radicals. The synergistic action of three enzymes can protect plants from free radical damage. When the concentration of heavy metals was low, the activity of protective enzymes increased under the induction of reactive oxygen radicals. However, with the increase of stress degree, the activities of SOD, POD and CAT decreased, which eventually led to the persecution of plant cells^34^. These conclusions are consistent with the results of this paper. When mine water was mixed with clear water at a ratio of 1:2, heavy metal stress stimulated the protective mechanism of ryegrass most appropriately, and improved plant quality and resistance. On the contrary, when the proportion of mine water increased, the physiological characteristics and quality of ryegrass plants were inhibited to different degrees.

Precious Nneka Amori et al. studied physiological traits of leaves and Silverbeet using treated wastewater, the results show that the biomass of plants watered with only the treated wastewater were more than 50% higher than the yield in tap water control and plants exhibited high degree of root foraging^1^. Libutti et al. irrigated tomato and broccoli with purified agro-industrial effluent and reported that yield and quality traits of agricultural products were not affected^35^. Radish was grown using a reclaimed synthetic textile wastewater treated in an anoxic-aerobic photobioreactor, and the dry weight, leaf number and leaf area of plant harvest were 49, 19.2 and 62% higher than the growth performance in freshwater irrigation^36^. FU et al. studied four native Chenopodiaceae plants of Halogeton glomeratus, Kochia scoparia, Suaeda glauca and Chenopodium glaucum in Jinchang area northwest China, from their changes of net photosynthetic rate (Pn), Stomatal conductance (Gs), transpiration rate (Tr). chlorophyll content (Chl), malondialdehyde (MDA), soluble protein (SP), proline (Pro) and antioxidant enzymes activity under the treatment of farmland soil (T1) and sedendary soil mixed with tailing (1:1, T2), they concluded that under T2 treatment, Pn, Gs, and Tr of Halogeton glomeratus and Kochia scoparia were decreased , the other six indexes were increased significantly. Gs, Tr, MDA, Pro, and SOD increased, yet CAT, Chl and Pn of Suaeda glauca decreased significantly, respectively. Pn, Gs, and Tr of Chenopodium glaucum decreased significantly, while SP, POD increased significantly^37^. Our results also indicated that mine water irrigation had significant effects on soil characteristics. At higher mine water ratios, the soil conductivity increased exponentially, the pH decreased gradually, the content of K^+^, Na^+^, Ca^2+^ and Mg^2+^ increased, and the content of N, P and K also increased gradually. In contrast, the clean water and mine water mixture had little effect on the soil properties. This was because the salt and metal ions in mine water migrate to the soil during the irrigation process, which significantly changes the soil properties. As a result, the concentration of salt in the soil increased and soil acidity also increased. After mixing with clean water, the concentration of salt decreases, and the influence on the soil matrix weakened. These results also indicated that the growth, physiological, and photosynthetic effects of ryegrass in the pot experiments were better than those in soilless culture, because there were many other organic materials and inorganic ions in soil that could promote growth, whereas the plants in the hydroponic system lacked other nutrients that benefit plant growth. Many existing studies have shown that mine drainage or other wastewater can improve the quality and yield of one or more kinds of plants to different degrees after certain treatment, but some studies also show that the reclaimed water used for irrigation will cause harm to plants, soil and even human health.

Jinfang Yang et al. reported that long-term irrigation with mine water significantly reduced the soil respiration rate and soil enzyme activity. Mine water irrigation also significantly inhibited wheat plant height, leaf area, chlorophyll content, and photosynthetic rate, and wheat production was also markedly reduced^38^. Jianjun Cha found that acidic mining waste water can reduce the pH of the soil profile and increase its electrical conductivity^39^. Junhao Qin et al. found that if treated mine water is used as an irrigation water source, acidic substances may still be introduced into the soil. This inhibits plant growth and may also enhance leaching of some trace elements in the soil to shallow aquifers, resulting in groundwater pollution^40, 41^. The results of this study are consistent with the above conclusions, that is, directly irrigating with mine water can significantly inhibit plant growth and photosynthesis, thus affecting the quality of ryegrass plants. MA et al. studied the effects of irrigation with mine wastewater on the physiological characters and heavy metals accumulation of winter wheat. It shows that irrigation with mine wastewater had negative effects on the winter wheat growth and grain yield. At anthesis stage, the leaf area, dry mass per stem, root activity and net photosynthetic rate of winter wheat in treatments were significantly lower and the plant height and leaf chlorophyll content was decreased. In addition, the heavy metals (Cr, Pb, Cu and Zn) contents in the grain of winter wheat under mine wastewater irrigation were significantly higher than those in control, it suggested that the irrigation with mine water could result in the heavy metals accumulation in wheat grain^42^. A large number of studies have shown that direct use of mine water for irrigation will have a negative impact on soil and plants, but this study found that after a certain processing of mine water (mine water was mixed with clear water in a ratio of 1:2) used for irrigation does not significantly alter soil properties, but can increase plant yield and quality, it will be meaningful to mine water reuse, soil utilization around the mining area and the agriculture.

The conclusions of this study are based on mine water from Fushun mining area in Northeast China, but the effects of mine water on plants from other mining areas are uncertain. At the same time, ryegrass, a cold-season turfgrass, is only selected in this study. If it is other kinds of plants, how they respond to mine water irrigation needs further study. What are the effects of mine water irrigation on plants other than ryegrass that need further study. Moreover, this study was only a short-term experiment, and the effects of mine water on the properties of the soil matrix cannot be generalized. Indoor experiments can be regularly watered to maintain moisture, indoor temperature is relatively fixed, while the natural environment is a lot of uncertainty and uncontrollable. Would the results of a small-scale pot experiment in a controlled environment be different if it were applied to a field where there are many uncertainties about soil properties and atmospheric conditions? Long-term field experiments must also be conducted to confirm our findings in more realistic conditions. The use of mine water resources not only has environmental and social benefits but could also bring economic benefits^43^. This study demonstrated that mine water can be used in ecological restoration and agricultural irrigation in mining areas, and is therefore of great significance to environmental restoration.

## Conclusion

Mine water is mostly groundwater leakage around coal seam, itself comes from groundwater. And the mining process will not produce a large number of organic or inorganic pollution substances. Therefore, compared with other industrial wastewater, mine water is a kind of water with high quality and relatively safe. Therefore, it is of great significance to use mine water for agricultural irrigation after proper treatment. Our findings indicated that soaking in mine water can prolong the germination time of ryegrass seeds but their germination rate was not significantly affected. When the ratio of clean water to mine water was 2:1, the germination potential and germination rate were the highest. Irrigating with mine water inhibited the physiological characteristics and photosynthetic characteristics of ryegrass. In contrast, mixing clean water with mine water for irrigation promoted ryegrass growth and photosynthesis. The promoting effect decreases with the increase of the proportion of mine water, and this effect was most significant when the ratio was 2:1. By analyzing the changes in the soil matrix properties, our study demonstrated that increasing the mine water ratio also increased the content of nutrient elements and metal cations in soil, as well as the electrical conductivity. Furthermore, the soil pH decreased, the soil salt content increased, and the acidity increased gradually. The mixing ratio of clean water and mine water at 2:1 had the least effects on soil matrix properties. Based on a comprehensive analysis of the influence of mine water irrigation on ryegrass and soil matrix, our study determined that irrigation with clean water and mine water at a 2:1 ratio could promote the growth and photosynthetic effect of ryegrass while also preserving soil quality. Therefore, this mixed irrigation ratio was considered optimal about the mine water from Laohutai mining area.

## Data Availability

The datasets used and/or analyzed in this study are available from the corresponding author upon reasonable request.
